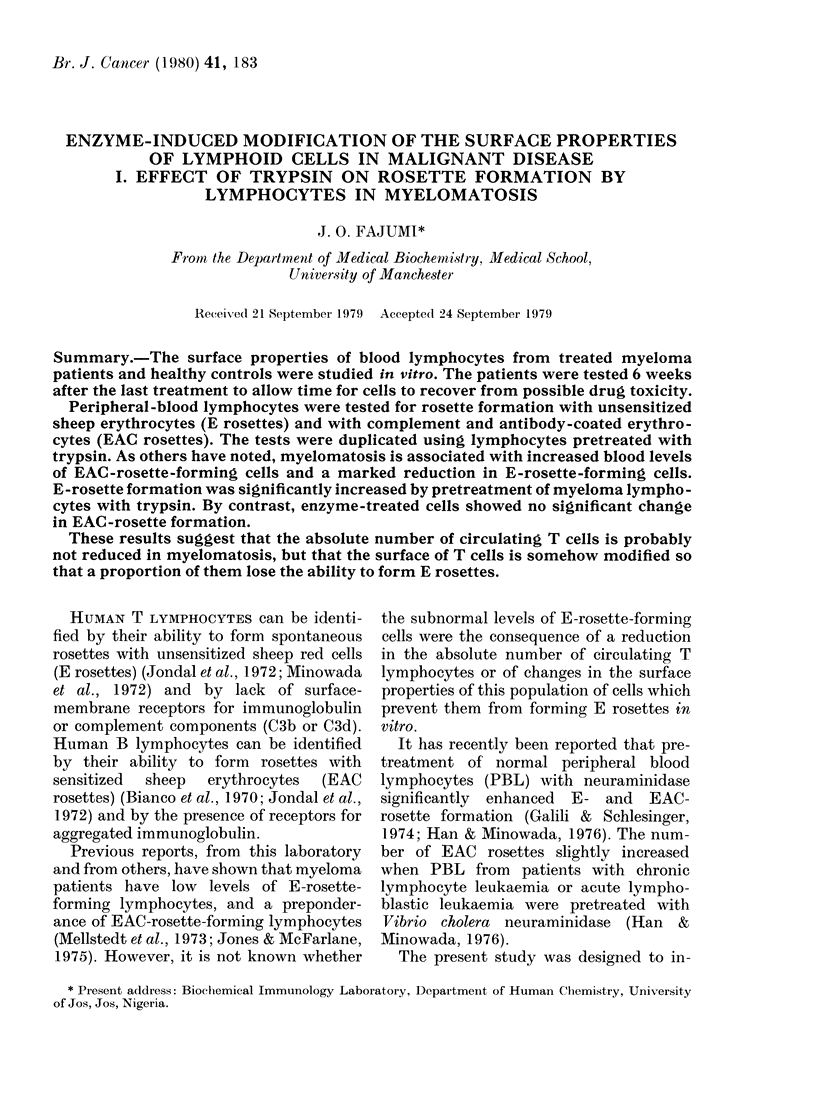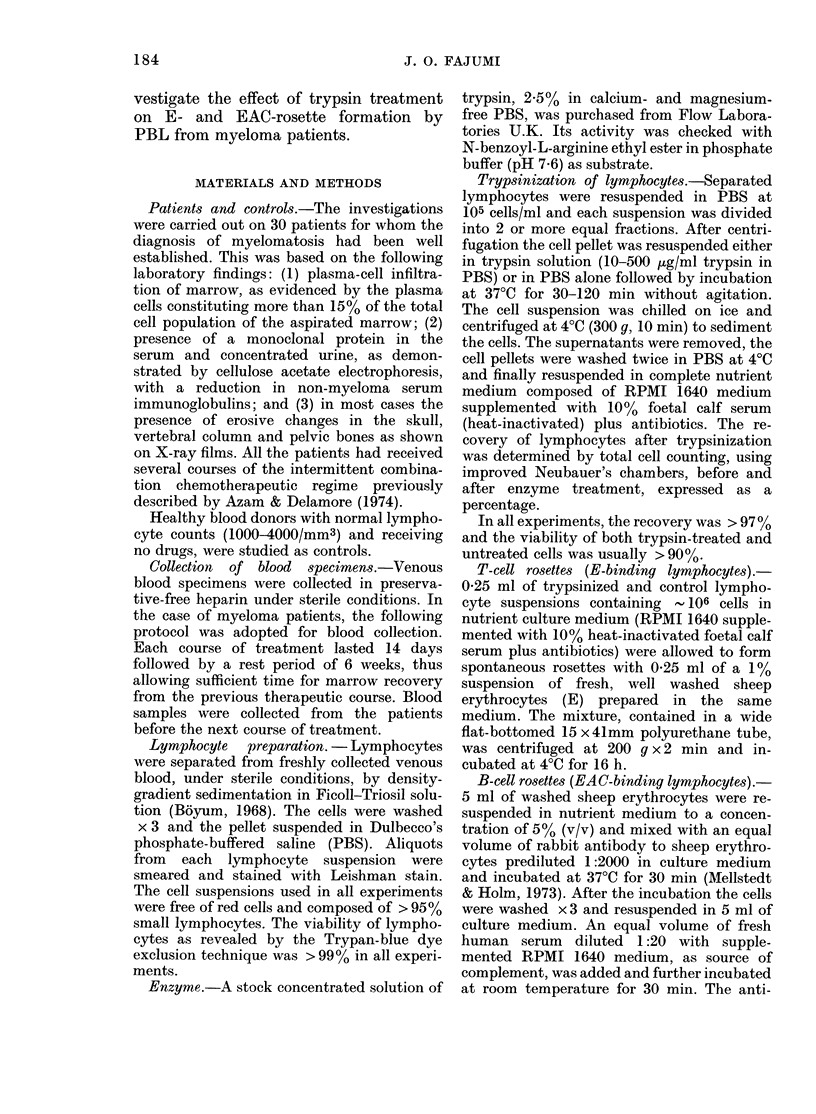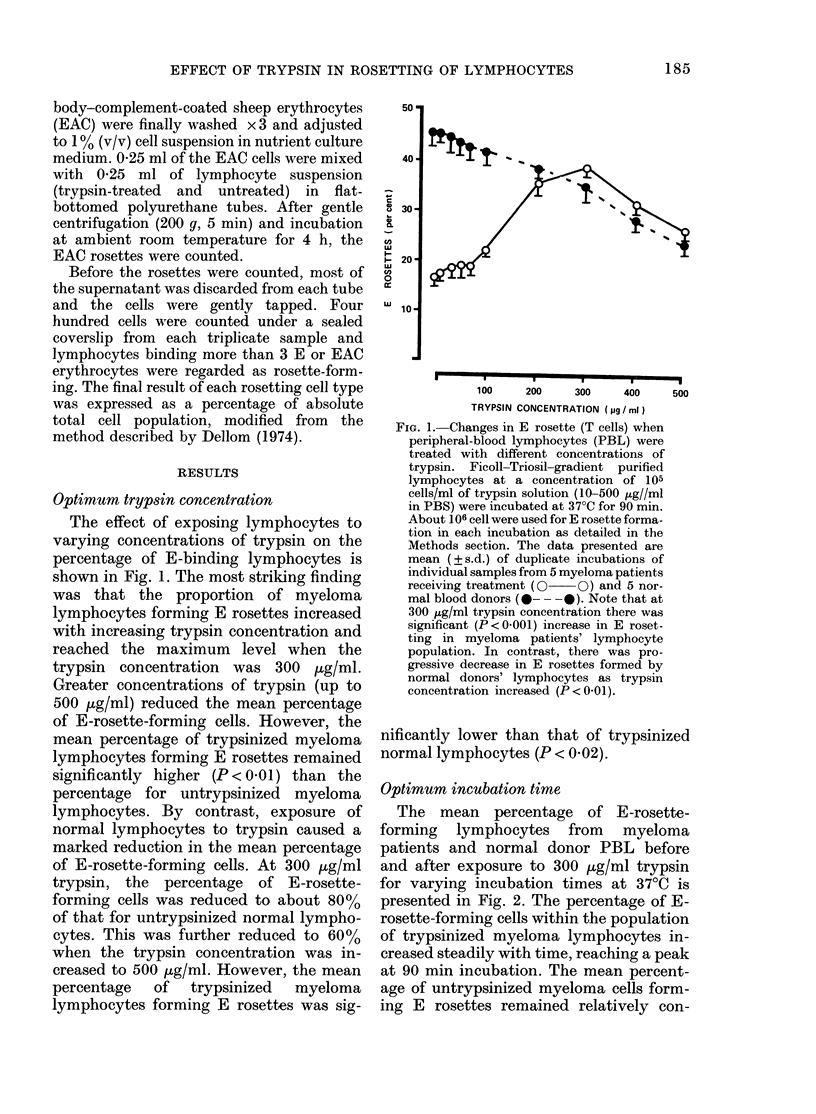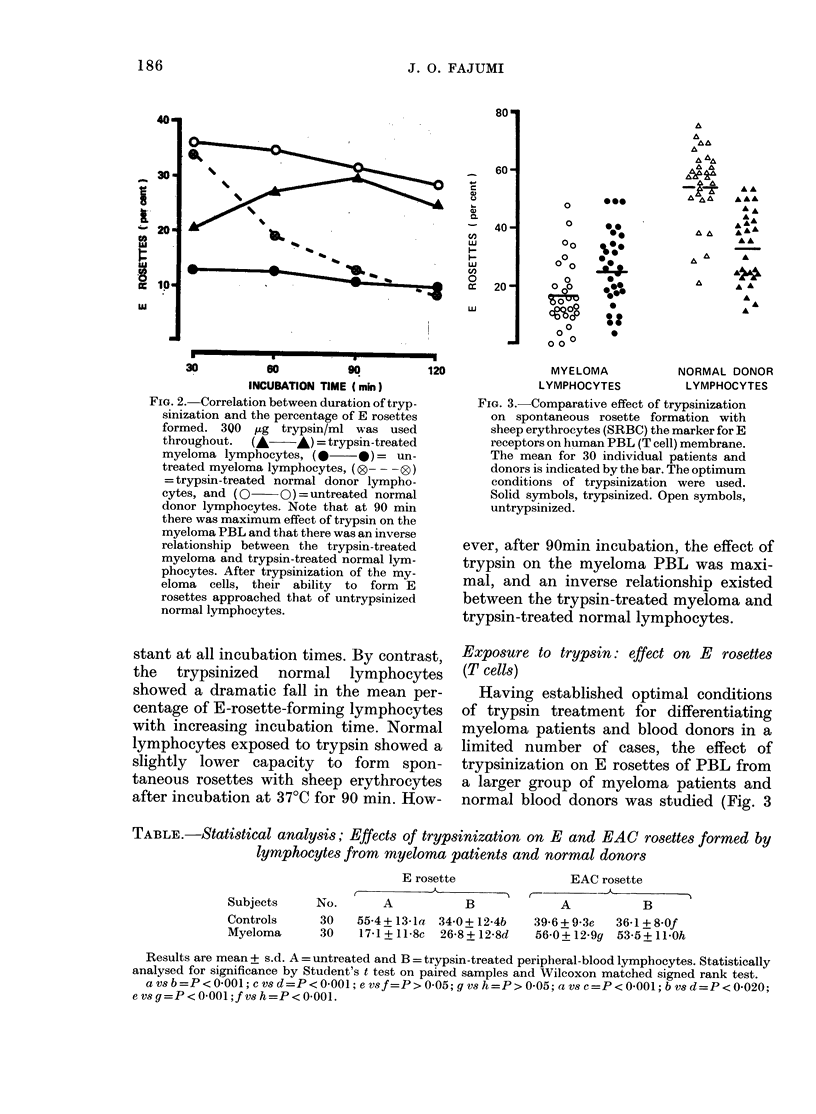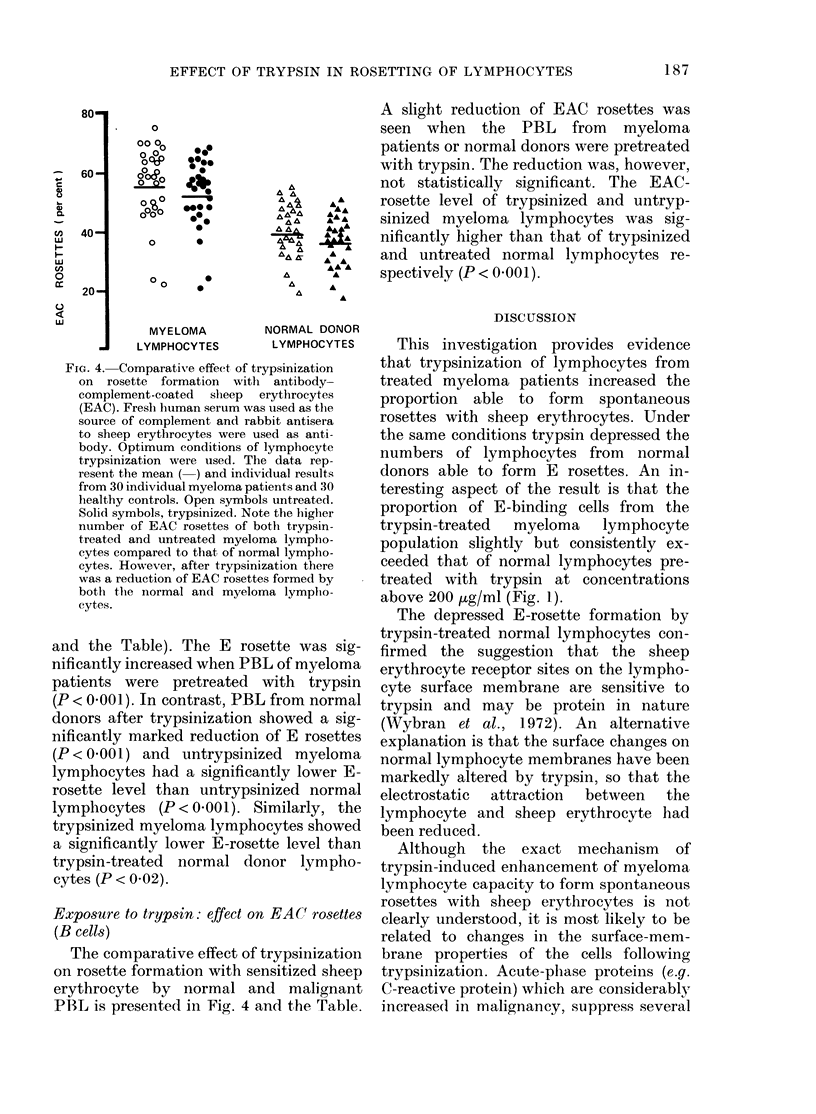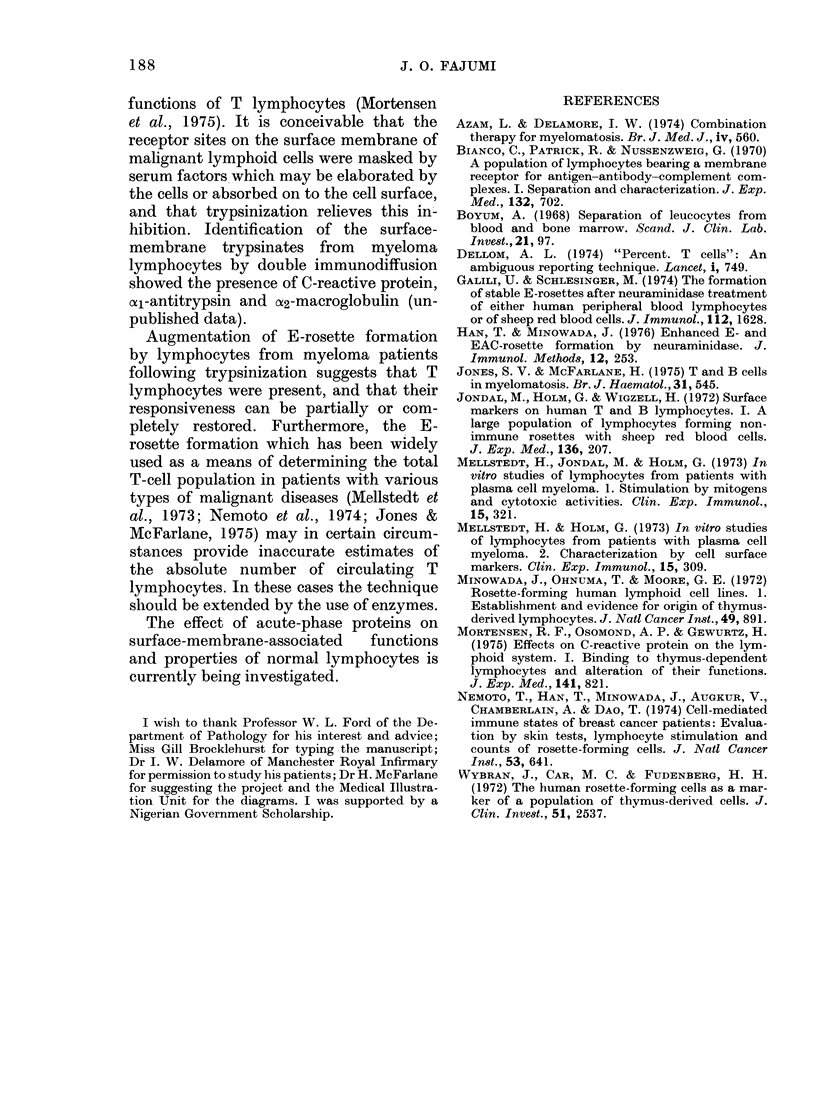# Enzyme-induced modification of the surface properties of lymphoid cells in malignant disease. I. Effect of trypsin on rosette formation by lymphocytes in myelomatosis.

**DOI:** 10.1038/bjc.1980.28

**Published:** 1980-02

**Authors:** J. O. Fajumi

## Abstract

The surface properties of blood lymphocytes from treated myeloma patients and healthy controls were studied in vitro. The patients were tested 6 weeks after the last treatment to allow time for cells to recovery from possible drug toxicity. Peripheral-blood lymphocytes were tested for rosette formation with unsensitized sheep erythrocytes (E rosettes) and with complement and antibody-coated erythrocytes (EAC rosettes). The tests were duplicated using lymphocytes pretreated with trypsin. As others have noted, myelomatosis is associated with increased blood levels of EAC-rosette-forming cells and a marked reduction in E-rosette-forming cells. E-rosette formation was significantly increased by pretreatment of myeloma lymphocytes with trypsin. By contrast, enzyme-treated cells showed no significant change in EAC-rosette formation. These results suggest that the absolute number of circulating T cells is probably not reduced in myelomatosis, but that the surface of T cells is somehow modified so that a proportion of them lose the ability to form E rosettes.


*Br. J. Cancer* (1980) **41**, 183

### ENZYME-INDUCED MODIFICATION OF THE SURFACE PROPERTIES OF LYMPHOID CELLS IN MALIGNANT DISEASE
### I. EFFECT OF TRYPSIN ON ROSETTE FORMATION BY LYMPHOCYTES IN MYELOMATOSIS

J. O. FAJUMI*

*From the Department of Medical Biochemistry, Medical School, University of Manchester*

Received 21 September 1979 Accepted 24 September 1979

**Summary.—The surface properties of blood lymphocytes from treated myeloma patients and healthy controls were studied *in vitro*. The patients were tested 6 weeks after the last treatment to allow time for cells to recover from possible drug toxicity.**

**Peripheral-blood lymphocytes were tested for rosette formation with unsensitized sheep erythrocytes (E rosettes) and with complement and antibody-coated erythrocytes (EAC rosettes). The tests were duplicated using lymphocytes pretreated with trypsin. As others have noted, myelomatosis is associated with increased blood levels of EAC-rosette-forming cells and a marked reduction in E-rosette-forming cells. E-rosette formation was significantly increased by pretreatment of myeloma lymphocytes with trypsin. By contrast, enzyme-treated cells showed no significant change in EAC-rosette formation.**

**These results suggest that the absolute number of circulating T cells is probably not reduced in myelomatosis, but that the surface of T cells is somehow modified so that a proportion of them lose the ability to form E rosettes.**

HUMAN T LYMPHOCYTES can be identified by their ability to form spontaneous rosettes with unsensitized sheep red cells (E rosettes) (Jondal *et al.*, 1972; Minowada *et al.*, 1972) and by lack of surface-membrane receptors for immunoglobulin or complement components (C3b or C3d). Human B lymphocytes can be identified by their ability to form rosettes with sensitized sheep erythrocytes (EAC rosettes) (Bianco *et al.*, 1970; Jondal *et al.*, 1972) and by the presence of receptors for aggregated immunoglobulin.

Previous reports, from this laboratory and from others, have shown that myeloma patients have low levels of E-rosette-forming lymphocytes, and a preponderance of EAC-rosette-forming lymphocytes (Mellstedt *et al.*, 1973; Jones & McFarlane, 1975). However, it is not known whether the subnormal levels of E-rosette-forming cells were the consequence of a reduction in the absolute number of circulating T lymphocytes or of changes in the surface properties of this population of cells which prevent them from forming E rosettes *in vitro*.

It has recently been reported that pretreatment of normal peripheral blood lymphocytes (PBL) with neuraminidase significantly enhanced E- and EAC-rosette formation (Galili & Schlesinger, 1974; Han & Minowada, 1976). The number of EAC rosettes slightly increased when PBL from patients with chronic lymphocyte leukaemia or acute lymphoblastic leukaemia were pretreated with *Vibrio cholera* neuraminidase (Han & Minowada, 1976).

The present study was designed to in-

* Present address: Biochemical Immunology Laboratory, Department of Human Chemistry, University of Jos, Jos, Nigeria.

vestigate the effect of trypsin treatment on E- and EAC-rosette formation by PBL from myeloma patients.

### MATERIALS AND METHODS

*Patients and controls.*—The investigations were carried out on 30 patients for whom the diagnosis of myelomatosis had been well established. This was based on the following laboratory findings: (1) plasma-cell infiltration of marrow, as evidenced by the plasma cells constituting more than 15% of the total cell population of the aspirated marrow; (2) presence of a monoclonal protein in the serum and concentrated urine, as demonstrated by cellulose acetate electrophoresis, with a reduction in non-myeloma serum immunoglobulins; and (3) in most cases the presence of erosive changes in the skull, vertebral column and pelvic bones as shown on X-ray films. All the patients had received several courses of the intermittent combination chemotherapeutic regime previously described by Azam & Delamore (1974).

Healthy blood donors with normal lymphocyte counts (1000–4000/mm$^3$) and receiving no drugs, were studied as controls.

*Collection of blood specimens.*—Venous blood specimens were collected in preservative-free heparin under sterile conditions. In the case of myeloma patients, the following protocol was adopted for blood collection. Each course of treatment lasted 14 days followed by a rest period of 6 weeks, thus allowing sufficient time for marrow recovery from the previous therapeutic course. Blood samples were collected from the patients before the next course of treatment.

*Lymphocyte preparation.* — Lymphocytes were separated from freshly collected venous blood, under sterile conditions, by density-gradient sedimentation in Ficoll–Triosil solution (Böyum, 1968). The cells were washed ×3 and the pellet suspended in Dulbecco's phosphate-buffered saline (PBS). Aliquots from each lymphocyte suspension were smeared and stained with Leishman stain. The cell suspensions used in all experiments were free of red cells and composed of >95% small lymphocytes. The viability of lymphocytes as revealed by the Trypan-blue dye exclusion technique was >99% in all experiments.

*Enzyme.*—A stock concentrated solution of trypsin, 2·5% in calcium- and magnesium-free PBS, was purchased from Flow Laboratories U.K. Its activity was checked with N-benzoyl-L-arginine ethyl ester in phosphate buffer (pH 7·6) as substrate.

*Trypsinization of lymphocytes.*—Separated lymphocytes were resuspended in PBS at $10^5$ cells/ml and each suspension was divided into 2 or more equal fractions. After centrifugation the cell pellet was resuspended either in trypsin solution (10–500 $\mu$g/ml trypsin in PBS) or in PBS alone followed by incubation at 37°C for 30–120 min without agitation. The cell suspension was chilled on ice and centrifuged at 4°C (300 *g*, 10 min) to sediment the cells. The supernatants were removed, the cell pellets were washed twice in PBS at 4°C and finally resuspended in complete nutrient medium composed of RPMI 1640 medium supplemented with 10% foetal calf serum (heat-inactivated) plus antibiotics. The recovery of lymphocytes after trypsinization was determined by total cell counting, using improved Neubauer's chambers, before and after enzyme treatment, expressed as a percentage.

In all experiments, the recovery was >97% and the viability of both trypsin-treated and untreated cells was usually >90%.

*T-cell rosettes (E-binding lymphocytes).*—0·25 ml of trypsinized and control lymphocyte suspensions containing ~$10^6$ cells in nutrient culture medium (RPMI 1640 supplemented with 10% heat-inactivated foetal calf serum plus antibiotics) were allowed to form spontaneous rosettes with 0·25 ml of a 1% suspension of fresh, well washed sheep erythrocytes (E) prepared in the same medium. The mixture, contained in a wide flat-bottomed 15×41mm polyurethane tube, was centrifuged at 200 *g*×2 min and incubated at 4°C for 16 h.

*B-cell rosettes (EAC-binding lymphocytes).*—5 ml of washed sheep erythrocytes were resuspended in nutrient medium to a concentration of 5% (v/v) and mixed with an equal volume of rabbit antibody to sheep erythrocytes prediluted 1:2000 in culture medium and incubated at 37°C for 30 min (Mellstedt & Holm, 1973). After the incubation the cells were washed ×3 and resuspended in 5 ml of culture medium. An equal volume of fresh human serum diluted 1:20 with supplemented RPMI 1640 medium, as source of complement, was added and further incubated at room temperature for 30 min. The antibody–complement-coated sheep erythrocytes (EAC) were finally washed ×3 and adjusted to 1% (v/v) cell suspension in nutrient culture medium. 0·25 ml of the EAC cells were mixed with 0·25 ml of lymphocyte suspension (trypsin-treated and untreated) in flat-bottomed polyurethane tubes. After gentle centrifugation (200 *g*, 5 min) and incubation at ambient room temperature for 4 h, the EAC rosettes were counted.

Before the rosettes were counted, most of the supernatant was discarded from each tube and the cells were gently tapped. Four hundred cells were counted under a sealed coverslip from each triplicate sample and lymphocytes binding more than 3 E or EAC erythrocytes were regarded as rosette-forming. The final result of each rosetting cell type was expressed as a percentage of absolute total cell population, modified from the method described by Dellom (1974).

### RESULTS

### *Optimum trypsin concentration*

The effect of exposing lymphocytes to varying concentrations of trypsin on the percentage of E-binding lymphocytes is shown in Fig. 1. The most striking finding was that the proportion of myeloma lymphocytes forming E rosettes increased with increasing trypsin concentration and reached the maximum level when the trypsin concentration was 300 μg/ml. Greater concentrations of trypsin (up to 500 μg/ml) reduced the mean percentage of E-rosette-forming cells. However, the mean percentage of trypsinized myeloma lymphocytes forming E rosettes remained significantly higher ($P < 0{\cdot}01$) than the percentage for untrypsinized myeloma lymphocytes. By contrast, exposure of normal lymphocytes to trypsin caused a marked reduction in the mean percentage of E-rosette-forming cells. At 300 μg/ml trypsin, the percentage of E-rosette-forming cells was reduced to about 80% of that for untrypsinized normal lymphocytes. This was further reduced to 60% when the trypsin concentration was increased to 500 μg/ml. However, the mean percentage of trypsinized myeloma lymphocytes forming E rosettes was significantly lower than that of trypsinized normal lymphocytes ($P < 0{\cdot}02$).

FIG. 1.—Changes in E rosette (T cells) when peripheral-blood lymphocytes (PBL) were treated with different concentrations of trypsin. Ficoll–Triosil-gradient purified lymphocytes at a concentration of $10^5$ cells/ml of trypsin solution (10–500 μg//ml in PBS) were incubated at 37°C for 90 min. About $10^6$ cell were used for E rosette formation in each incubation as detailed in the Methods section. The data presented are mean (±s.d.) of duplicate incubations of individual samples from 5 myeloma patients receiving treatment (○——○) and 5 normal blood donors (●- - -●). Note that at 300 μg/ml trypsin concentration there was significant ($P < 0{\cdot}001$) increase in E rosetting in myeloma patients' lymphocyte population. In contrast, there was progressive decrease in E rosettes formed by normal donors' lymphocytes as trypsin concentration increased ($P < 0{\cdot}01$).

### *Optimum incubation time*

The mean percentage of E-rosette-forming lymphocytes from myeloma patients and normal donor PBL before and after exposure to 300 μg/ml trypsin for varying incubation times at 37°C is presented in Fig. 2. The percentage of E-rosette-forming cells within the population of trypsinized myeloma lymphocytes increased steadily with time, reaching a peak at 90 min incubation. The mean percentage of untrypsinized myeloma cells forming E rosettes remained relatively con-

FIG. 2.—Correlation between duration of trypsinization and the percentage of E rosettes formed. 3Q0 μg trypsin/ml was used throughout. (▲——▲) = trypsin-treated myeloma lymphocytes, (●——●) = untreated myeloma lymphocytes, (⊗- - -⊗) = trypsin-treated normal donor lymphocytes, and (○——○) = untreated normal donor lymphocytes. Note that at 90 min there was maximum effect of trypsin on the myeloma PBL and that there was an inverse relationship between the trypsin-treated myeloma and trypsin-treated normal lymphocytes. After trypsinization of the myeloma cells, their ability to form E rosettes approached that of untrypsinized normal lymphocytes.

FIG. 3.—Comparative effect of trypsinization on spontaneous rosette formation with sheep erythrocytes (SRBC) the marker for E receptors on human PBL (T cell) membrane. The mean for 30 individual patients and donors is indicated by the bar. The optimum conditions of trypsinization were used. Solid symbols, trypsinized. Open symbols, untrypsinized.

stant at all incubation times. By contrast, the trypsinized normal lymphocytes showed a dramatic fall in the mean percentage of E-rosette-forming lymphocytes with increasing incubation time. Normal lymphocytes exposed to trypsin showed a slightly lower capacity to form spontaneous rosettes with sheep erythrocytes after incubation at 37°C for 90 min. However, after 90min incubation, the effect of trypsin on the myeloma PBL was maximal, and an inverse relationship existed between the trypsin-treated myeloma and trypsin-treated normal lymphocytes.

*Exposure to trypsin: effect on E rosettes (T cells)*

Having established optimal conditions of trypsin treatment for differentiating myeloma patients and blood donors in a limited number of cases, the effect of trypsinization on E rosettes of PBL from a larger group of myeloma patients and normal blood donors was studied (Fig. 3

TABLE.—*Statistical analysis; Effects of trypsinization on E and EAC rosettes formed by lymphocytes from myeloma patients and normal donors*

| | | E rosette | | EAC rosette | |
|---|---|---|---|---|---|
| Subjects | No. | A | B | A | B |
| Controls | 30 | 55·4 ± 13·1*a* | 34·0 ± 12·4*b* | 39·6 ± 9·3*e* | 36·1 ± 8·0*f* |
| Myeloma | 30 | 17·1 ± 11·8*c* | 26·8 ± 12·8*d* | 56·0 ± 12·9*g* | 53·5 ± 11·0*h* |

Results are mean ± s.d. A = untreated and B = trypsin-treated peripheral-blood lymphocytes. Statistically analysed for significance by Student's *t* test on paired samples and Wilcoxon matched signed rank test.
$a$ *vs* $b = P < 0{\cdot}001$; $c$ *vs* $d = P < 0{\cdot}001$; $e$ *vs* $f = P > 0{\cdot}05$; $g$ *vs* $h = P > 0{\cdot}05$; $a$ *vs* $c = P < 0{\cdot}001$; $b$ *vs* $d = P < 0{\cdot}020$; $e$ *vs* $g = P < 0{\cdot}001$; $f$ *vs* $h = P < 0{\cdot}001$.

FIG. 4.—Comparative effect of trypsinization on rosette formation with antibody–complement-coated sheep erythrocytes (EAC). Fresh human serum was used as the source of complement and rabbit antisera to sheep erythrocytes were used as antibody. Optimum conditions of lymphocyte trypsinization were used. The data represent the mean (—) and individual results from 30 individual myeloma patients and 30 healthy controls. Open symbols untreated. Solid symbols, trypsinized. Note the higher number of EAC rosettes of both trypsin-treated and untreated myeloma lymphocytes compared to that of normal lymphocytes. However, after trypsinization there was a reduction of EAC rosettes formed by both the normal and myeloma lymphocytes.

and the Table). The E rosette was significantly increased when PBL of myeloma patients were pretreated with trypsin ($P < 0{\cdot}001$). In contrast, PBL from normal donors after trypsinization showed a significantly marked reduction of E rosettes ($P < 0{\cdot}001$) and untrypsinized myeloma lymphocytes had a significantly lower E-rosette level than untrypsinized normal lymphocytes ($P < 0{\cdot}001$). Similarly, the trypsinized myeloma lymphocytes showed a significantly lower E-rosette level than trypsin-treated normal donor lymphocytes ($P < 0{\cdot}02$).

*Exposure to trypsin: effect on EAC rosettes (B cells)*

The comparative effect of trypsinization on rosette formation with sensitized sheep erythrocyte by normal and malignant PBL is presented in Fig. 4 and the Table. A slight reduction of EAC rosettes was seen when the PBL from myeloma patients or normal donors were pretreated with trypsin. The reduction was, however, not statistically significant. The EAC-rosette level of trypsinized and untrypsinized myeloma lymphocytes was significantly higher than that of trypsinized and untreated normal lymphocytes respectively ($P < 0{\cdot}001$).

### DISCUSSION

This investigation provides evidence that trypsinization of lymphocytes from treated myeloma patients increased the proportion able to form spontaneous rosettes with sheep erythrocytes. Under the same conditions trypsin depressed the numbers of lymphocytes from normal donors able to form E rosettes. An interesting aspect of the result is that the proportion of E-binding cells from the trypsin-treated myeloma lymphocyte population slightly but consistently exceeded that of normal lymphocytes pretreated with trypsin at concentrations above 200 μg/ml (Fig. 1).

The depressed E-rosette formation by trypsin-treated normal lymphocytes confirmed the suggestion that the sheep erythrocyte receptor sites on the lymphocyte surface membrane are sensitive to trypsin and may be protein in nature (Wybran *et al.*, 1972). An alternative explanation is that the surface changes on normal lymphocyte membranes have been markedly altered by trypsin, so that the electrostatic attraction between the lymphocyte and sheep erythrocyte had been reduced.

Although the exact mechanism of trypsin-induced enhancement of myeloma lymphocyte capacity to form spontaneous rosettes with sheep erythrocytes is not clearly understood, it is most likely to be related to changes in the surface-membrane properties of the cells following trypsinization. Acute-phase proteins (*e.g.* C-reactive protein) which are considerably increased in malignancy, suppress several functions of T lymphocytes (Mortensen *et al.*, 1975). It is conceivable that the receptor sites on the surface membrane of malignant lymphoid cells were masked by serum factors which may be elaborated by the cells or absorbed on to the cell surface, and that trypsinization relieves this inhibition. Identification of the surface-membrane trypsinates from myeloma lymphocytes by double immunodiffusion showed the presence of C-reactive protein, $\alpha_1$-antitrypsin and $\alpha_2$-macroglobulin (unpublished data).

Augmentation of E-rosette formation by lymphocytes from myeloma patients following trypsinization suggests that T lymphocytes were present, and that their responsiveness can be partially or completely restored. Furthermore, the E-rosette formation which has been widely used as a means of determining the total T-cell population in patients with various types of malignant diseases (Mellstedt *et al.*, 1973; Nemoto *et al.*, 1974; Jones & McFarlane, 1975) may in certain circumstances provide inaccurate estimates of the absolute number of circulating T lymphocytes. In these cases the technique should be extended by the use of enzymes.

The effect of acute-phase proteins on surface-membrane-associated functions and properties of normal lymphocytes is currently being investigated.

I wish to thank Professor W. L. Ford of the Department of Pathology for his interest and advice; Miss Gill Brocklehurst for typing the manuscript; Dr I. W. Delamore of Manchester Royal Infirmary for permission to study his patients; Dr H. McFarlane for suggesting the project and the Medical Illustration Unit for the diagrams. I was supported by a Nigerian Government Scholarship.

### REFERENCES

AZAM, L. & DELAMORE, I. W. (1974) Combination therapy for myelomatosis. *Br. J. Med. J.*, **iv**, 560.

BIANCO, C., PATRICK, R. & NUSSENZWEIG, G. (1970) A population of lymphocytes bearing a membrane receptor for antigen–antibody–complement complexes. I. Separation and characterization. *J. Exp. Med.*, **132**, 702.

BOYUM, A. (1968) Separation of leucocytes from blood and bone marrow. *Scand. J. Clin. Lab. Invest.*, **21**, 97.

DELLOM, A. L. (1974) "Percent. T cells": An ambiguous reporting technique. *Lancet*, **i**, 749.

GALILI, U. & SCHLESINGER, M. (1974) The formation of stable E-rosettes after neuraminidase treatment of either human peripheral blood lymphocytes or of sheep red blood cells. *J. Immunol.*, **112**, 1628.

HAN, T. & MINOWADA, J. (1976) Enhanced E- and EAC-rosette formation by neuraminidase. *J. Immunol. Methods*, **12**, 253.

JONES, S. V. & MCFARLANE, H. (1975) T and B cells in myelomatosis. *Br. J. Haematol.*, **31**, 545.

JONDAL, M., HOLM, G. & WIGZELL, H. (1972) Surface markers on human T and B lymphocytes. I. A large population of lymphocytes forming non-immune rosettes with sheep red blood cells. *J. Exp. Med.*, **136**, 207.

MELLSTEDT, H., JONDAL, M. & HOLM, G. (1973) *In vitro* studies of lymphocytes from patients with plasma cell myeloma. 1. Stimulation by mitogens and cytotoxic activities. *Clin. Exp. Immunol.*, **15**, 321.

MELLSTEDT, H. & HOLM, G. (1973) *In vitro* studies of lymphocytes from patients with plasma cell myeloma. 2. Characterization by cell surface markers. *Clin. Exp. Immunol.*, **15**, 309.

MINOWADA, J., OHNUMA, T. & MOORE, G. E. (1972) Rosette-forming human lymphoid cell lines. 1. Establishment and evidence for origin of thymus-derived lymphocytes. *J. Natl Cancer Inst.*, **49**, 891.

MORTENSEN, R. F., OSOMOND, A. P. & GEWURTZ, H. (1975) Effects on C-reactive protein on the lymphoid system. I. Binding to thymus-dependent lymphocytes and alteration of their functions. *J. Exp. Med.*, **141**, 821.

NEMOTO, T., HAN, T., MINOWADA, J., AUGKUR, V., CHAMBERLAIN, A. & DAO, T. (1974) Cell-mediated immune states of breast cancer patients: Evaluation by skin tests, lymphocyte stimulation and counts of rosette-forming cells. *J. Natl Cancer Inst.*, **53**, 641.

WYBRAN, J., CAR, M. C. & FUDENBERG, H. H. (1972) The human rosette-forming cells as a marker of a population of thymus-derived cells. *J. Clin. Invest.*, **51**, 2537.